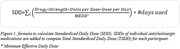# Relationship between Anticholinergics and Cognitive Measures Important to the Diagnosis of Dementia

**DOI:** 10.1002/alz.095389

**Published:** 2025-01-09

**Authors:** Noha Keshk, Hussein Khalil, Noll L Campbell

**Affiliations:** ^1^ Purdue University, West Lafayette, IN USA; ^2^ Mansoura University, Mansoura, Dakahliya Egypt; ^3^ Indiana University Center for Aging Research, Indianapolis, IN USA

## Abstract

**Background:**

Over half of ambulatory older adults are prescribed at least one anticholinergic medication to address a myriad of conditions, including incontinence, allergies, anxiety and insomnia. Longitudinal studies suggest an association between anticholinergic exposure and risk of dementia. Prior studies often rely on crude exposure measures (use vs. non‐use), lacking detailed analysis of anticholinergic effects across different cognitive domains indicative of dementia. This study adopts a more comprehensive exposure measure, considering dosage, duration, and strength, to explore its association with a spectrum of cognitive assessments commonly used in diagnosing dementia.

**Method:**

A retrospective observational design was employed, analyzing one‐year electronic prescription data of 735 participants (age, 70.12±6.33; 75.2% Female; 88.3% White) recruited in two clinical trials (BrainSafe and R2D2 studies). Anticholinergic exposure was quantified using total standardized daily doses (TSDD). Outcomes were cognitive test scores of phonemic fluency, semantic fluency, symbol digit modalities, oral trail making (OTMT Part A and B), and hopkins verbal learning test (HVLT) collected at baseline. Generalized Linear models were applied to evaluate the impact of TSDD on cognitive performance, and Least Square means with Tukey’s adjustment were utilized to compare effects across anticholinergic subclasses. Subclasses compared were antimuscarinics, antihistamines, skeletal muscle relaxants and antidepressants. Covariates included in all the models are age, gender, ethnicity, educational level, and Charlson Co‐morbidity Index (CCI) Scores.

**Result:**

Higher anticholinergic exposure was associated with poorer performance in semantic fluency test (p‐value = 0.0002) and OTMT‐A (p‐value = 0.0034). The comparison between anticholinergic subclasses showed that antimuscarinic only users performed significantly better in HVLT total recall test than those using a combination of antihistamines and antimuscarinics (p‐value = 0.0037), after adjusting for TSDD and other covariates. Both antihistamine users and antimuscarinic users exhibited worse outcomes than antidepressant users in semantic fluency (p‐value = 0.008 and p‐value = 0.005, respectively).

**Conclusion:**

This study indicates an adverse dose‐duration effect of anticholinergic drugs on language, complex attention, executive functioning, and processing speed. Specifically, antimuscarinics users and antihistamine users demonstrated inferior performance in language, processing speed, and executive function compared to those on antidepressants. Additionally, combination use of antihistamines and antimuscarinics may lead to worse memory recall compared to the use of antimuscarinics alone.